# Development of Highly Repellent Silica Particles for Protection of Hemp Shiv Used as Insulation Materials

**DOI:** 10.3390/ma11010004

**Published:** 2017-12-21

**Authors:** Marion A. Bourebrab, Géraldine G. Durand, Alan Taylor

**Affiliations:** 1 National Structure Integrity Research Centre, Granta Park, Great Abington, Cambridge CB21 6AL, UK; 2 School of Engineering, The University of Edinburgh, Edinburgh EH9 3FB, UK; 3 TWI Ltd., Granta Park, Great Abington, Cambridge CB21 6AL, UK; durandg@lsbu.ac.uk (G.G.D.); alan.taylor@twi.co.uk (A.T.); 4 Advanced Resins and Coatings Technologies Innovation Centre, School of Engineering, London South Bank University, 103 Borough Road, London SE1 0AA, UK

**Keywords:** hemp shiv, insulation, hydrophobicity, silica particles, stöber process

## Abstract

New bio-materials have recently gained interest for use in insulation panels in walls, but wider adoption by the building industry is hindered by their intrinsic properties. The fact that such materials are mainly composed of cellulose makes them combustible, and their hydrophilic surface presents a high water uptake, which would lead to faster biodegradation. A hydrophobic treatment with silica particles was successfully synthesised via Stöber process, characterised, and deposited on hemp shiv. The surface of hemp shiv coated several times with 45 and 120 nm particles were uniformly covered, as well as extensively water repellent. Those samples could withstand in humidity chamber without loss of their hydrophobic property and no sign of mould growth after 72 h of exposure.

## 1. Introduction

Hemp plant (*Cannabis sativa* L.) has recently drawn much interest in the construction sector for the manufacture of bio-based composite structures [1,2,3,4]. The plant does not require a lot of water to grow, is easy to harvest in moderate climates [5], and exhibits a range of interesting and useful properties. The woody core of the plant, usually named hemp shiv, is very porous with a low bulk density [4], which makes it a valuable candidate for use as an insulation material for both acoustic [6,7] and thermal [8,9,10,11] applications.

The porosity of hemp provides the ability to create a breathable structure, where moisture can be absorbed and desorbed from its surrounding environment [3]. This characteristic enables such insulation to act as a buffer that regulates against sudden changes in temperature and humidity. One recent study demonstrated this effect by comparing the energy transfer efficiency of a hemp-lime wall assembly and a conventional mineral wool one when subjected to a sudden temperature drop. The hemp wall transferred 17% of energy compared to 75% for the mineral wool one [12]. The hemp-based insulating material thus better regulated the indoor air environment by moderating changes, which is likely to translate to reduced need for air conditioning, and ultimately energy savings. Extensive research has been carried out with similar hemp-lime wall assemblies for internal partitions [3,7,12,13,14,15,16,17,18]. In the example of a hemp-lime assembly, protection against biodegradation is offered via the highly alkaline lime present [18]. However, the protection of the hemp shiv only is a novel field of study, with industrial impacts.

The objective of the ISOBIO project, under which the work reported here was carried out, is to develop low embodied energy bio-based insulation materials, for use in building envelopes either for new build or for retrofitting to existing buildings.

Even though there are drivers to use more ecologically responsible materials in the construction sector, their composition still hinders a widespread usage. For example, hemp shiv are made from 40–52% cellulose, 22–30% lignin, and 18–28% hemicellulose, as well as pectins, ashes, waxes, and fat in smaller quantities [8,19]. The relative quantities vary considerably depending on how the hemp was harvested, the soil, the climatic conditions, etc. Nevertheless, the primary constituents are hydrocarbons and so are combustible, whilst the hydroxyl groups of the cellulose parts are hydrophilic. It becomes crucial to address these two issues if bio-materials are the main constituent of a building block.

Another group of researchers under the same project have successfully demonstrated that hemp shiv could be chemically modified to become hydrophobic, by the means of a sol-gel derived, silica based thin mesoporous coating [20]. In common with many alkoxide sol-gel systems, this is an evolving dynamic system with a certain shelf-life and can be prone to gelling. Our approach differs from theirs in that hydrophobicity is introduced by depositing hydrophobic silica particles onto the hemp shiv. These particles are already fully reacted by the time of deposition. There are two reasons behind using inorganic material on the bio-aggregates: different sizes of silica particles can be deposited giving coatings of different thicknesses; and having inorganic silica creates a thermal shield that would then help dissipate heat when the material would be exposed to fire. The authors’ future work is focussed on this dual functionality of the silica particles. This study thus focusses on applying functionalised silica particles onto hemp shiv to provide hydrophobic properties and prevent degradation in humid environment, so that those bio-materials could be effectively used as insulation materials in building envelopes.

## 2. Materials and Methods

The approach followed in this study was to develop a hydrophobic treatment to be applied on bio-based insulation materials, in particular hemp shiv. Silica particles were synthesised and functionalised to repel water, then applied directly on the shiv, according to the processes described below.

### 2.1. Materials

Silica particles were synthesised using sol-gel methods with tetraethoxysilane (TEOS) as the silica precursor (Silanes & Silicones, Stockport, UK). Ammonium hydroxide (Sigma-Aldrich, Gillingham, UK) was diluted in-house from 28–30 wt % to 25 wt %. Industrial methylated spirit (IMS) (99% ethanol, 1% methanol), supplied by VWR International (Lutterworth, UK), was used as the co-solvent. Finally, water was deionised in-house. *N*-propyl trimethoxysilane (nPTMS) was used as the functionalising agent (Silanes & Silicones, Stockport, UK) with dibutyltin dilaurate (Sigma-Aldrich, Gillingham, UK) as the condensation catalyst [21].

Different size distributions of hemp shiv were kindly provided by Cavac Biomatériaux (Sainte-Gemme-La-Plaine, France): the smallest being Isofin (around 2 mm in length), then G7 (7 mm), G8 (8 mm), Biofibat (10 mm), and G14 (14 mm).

### 2.2. Silica Particles Synthesis

In this study, all the silica particles were synthesised via the Stöber process [22], at room temperature, according to the authors’ previous work [23]. Three formulations of silica particles were synthesised: one that led to a particle diameter size of approximately 30 nm, and 45 nm when functionalised; one with a functionalised particle size of approximately 120 nm; and one with a functionalised particle size of 380 nm. All distributions were uniform and mono-modal (with a polydispersity index below 0.2), and formulated to reach 4.3 wt % silica at the end of the reaction. The ammonia was then removed from the systems by evaporation using a Rotovap and IMS was added so that the final content remained at 4.3 wt % silica.

### 2.3. Functionalisation

Silica surfaces are typically hydroxyl rich, silica nanoparticles made via precipitation or pyrogenic methods have silanol densities between 4 and 6 OH/nm^2^ [24]. In the presence of a catalyst, alkoxysilanes are grafted onto the particle surfaces by reaction with the silanol groups (Si-OH) [25,26,27]. Having alkyl groups grafted on the particles gives two advantages: the silica particles are transformed from hydrophilic to hydrophobic, and the particles are prevented from aggregating due to sterical stabilisation. In this study, n-propyl trimethoxysilane (nPTMS) was reacted with the silica particles dispersed in alcohol via the use of the tin catalyst by heating at 65 °C for 18 h to allow hydrolysis-free condensation reaction between the alkoxy silane and silanol rich surfaces of the silica particles [21]. The relative quantities of the silane and the silica were varied to assess the impact of the silane. A simple gravimetric functionalisation ratio was defined as the quantity of silane as a function of the quantity of silica in the suspension, following the example of Posthumus et al. [28].

### 2.4. Deposition

Silica particles were deposited onto glass slides to allow accurate sessile drop assessment without the influence of the roughness of the hemp shiv. Coatings were prepared by immersing soda-lime glass microscope slides into the silica suspension and withdrawing at 100 mm/min. The glass slides were dried at 150 °C for one hour to remove all volatiles including excess unreacted silane.

Prior to coating, the hemp shiv samples were dried in the oven at 90 °C for one hour. They were then immersed in a dispersion of silica particles for two minutes with constant mixing, to ensure all aggregates were coated by the silica particles. The bio-materials were then strained to remove all the liquid, and dried in the oven at 90 °C for one hour. This process was repeated to build up multiple layers of silica particles.

The hemp shiv samples prepared are specified in Table 1. The formulations used for the series of samples H4-x correspond to dispersions of functionalised 45 nm silica particles; samples H12-x with particles of 120 nm diameter; and H38-x to those treated with 380 nm diameter silica particles.

### 2.5. Characterisation

The protocol used for characterising the different samples was as follows:

- Analysis of the particle size distributions (mean diameter (Z average) and polydispersity index values measured) of the silica suspensions;
- Dip-coating of three glass slides for each formulation followed by drying at 150 °C for one hour;
- Water contact angle (WCA) measurements on glass slides, to verify the hydrophobic character of the dispersion (WCA averaged from measurements of three to ten droplets of water on each of the three glass slides);
- Coating via immersion of hemp shiv and drying at 90 °C for one hour;
- Water contact angle measurements on hemp shiv, and time for which the WCA remains greater than 90°;
- Imaging of samples via SEM and EDX characterisation; and
- Analysis of the environmental conditions on the treated hemp shiv: immersion in a water bath for 24 h/test in humidity chamber at 35 °C/90% relative humidity for 72 h, followed by drying and WCA measurements.

**Imaging via Scanning Electron Microscopy (SEM):** Examination of the surface of coated hemp shiv was undertaken using SEM techniques. The samples were gold coated using sputtering methods prior to analysis. The images of coated hemp shiv were produced on a Zeiss Σigma FEGSEM (focussed electron gun SEM) (Zeiss, Germany) operating at 5 kV, using a secondary electron detector for topography analysis.

**Energy-dispersive X-ray (EDX) spectroscopy:** Elemental analysis of the coated surfaces was undertaken by obtaining EDX spectra of the selected areas of the samples and analysed via the AZtec characterisation system (Oxford Instruments, UK).

**Figure 1:**
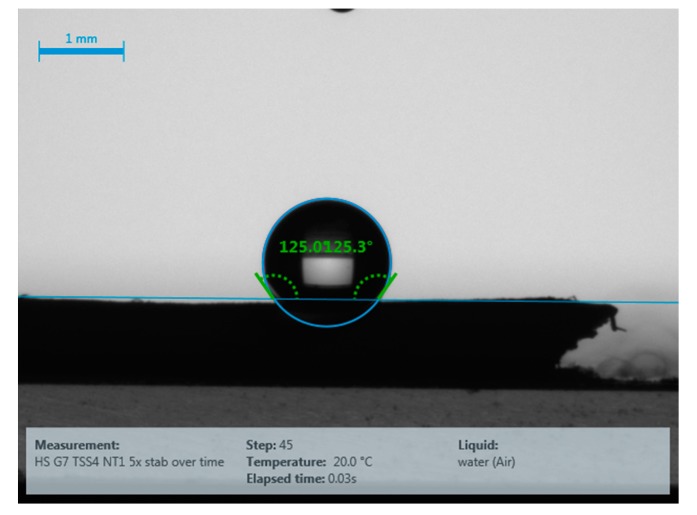
DSA image captured via the Krüss Advance software, with the example of sample H4-1-5. Courtesy of TWI Ltd.

**Water contact angle (WCA) measurement:** The hydrophobic character of the samples was assessed by measuring static sessile drop characteristics. In this study, all static water contact angle measurements were performed using a Drop Shape Analyser DSA-100 (Krüss GmbH, Hamburg, Germany). For all materials, between three and ten 2 μL droplets of deionised water were placed onto the samples, images of the droplets were captured allowing key dimensions to be measured. This data was analysed using Krüss Advance software (Figure 1). The average WCA was then reported, as well as the standard deviation for each formulation of treatment applied on the hemp shiv. The measurements were performed at room temperature (24 ± 1 °C).

**Humidity exposure:** The durability of the treatment in a humid environment was assessed by placing loose coated hemp shiv in a humidity chamber (Vötsch VC 0020, Weiss Technik, Germany). There is currently no benchmark test to assess the durability of a hydrophobic coating deposited on bio-materials in humid environment. It was decided to set the parameters of the humidity chamber at 35 °C and 90% relative humidity (RH) according to recommendations from the ISOBIO project. The materials were taken out every 24 h to measure the WCA to monitor any loss of water repellence due to the environment. The test was terminated when traces of mould growth were visible on at least one sample. The undamaged samples were dried at room temperature for 5 days.

## 3. Results

### 3.1. Assessment of Functionalisation

**Figure 2:**
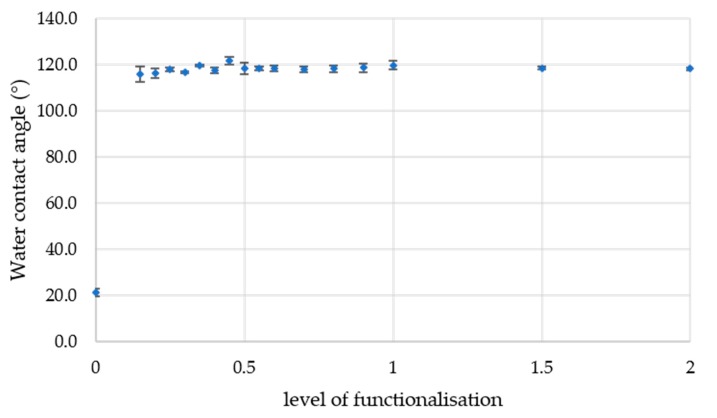
Water contact angle measurements (the standard deviation is shown as error bars) on glass slides.

The dispersion of unfunctionalised particles deposited as a single layer on glass slides gave a water contact angle of 21°, demonstrating a hydrophilic character. All other formulations of nPTMS-functionalised silica had water contact angles of 118.5 ± 0.8°. This is presented graphically in Figure 2. The quantity of silane used did not appear to have a significant influence on the repellence to water when measured on glass slides.

### 3.2. Uniformity of Deposition

Nine samples of hemp shiv, without and with surface treatment with silica particles (H0-0-0, H4-1-x, and H12-1-x) were examined using SEM imaging. Several samples of hemp shiv coated with the same formulation were imaged, at four magnifications: ×1000, ×5000, ×10,000, and ×25,000. This allowed a representative overview of how uniform the deposition of each coating was.

**Figure 3:**
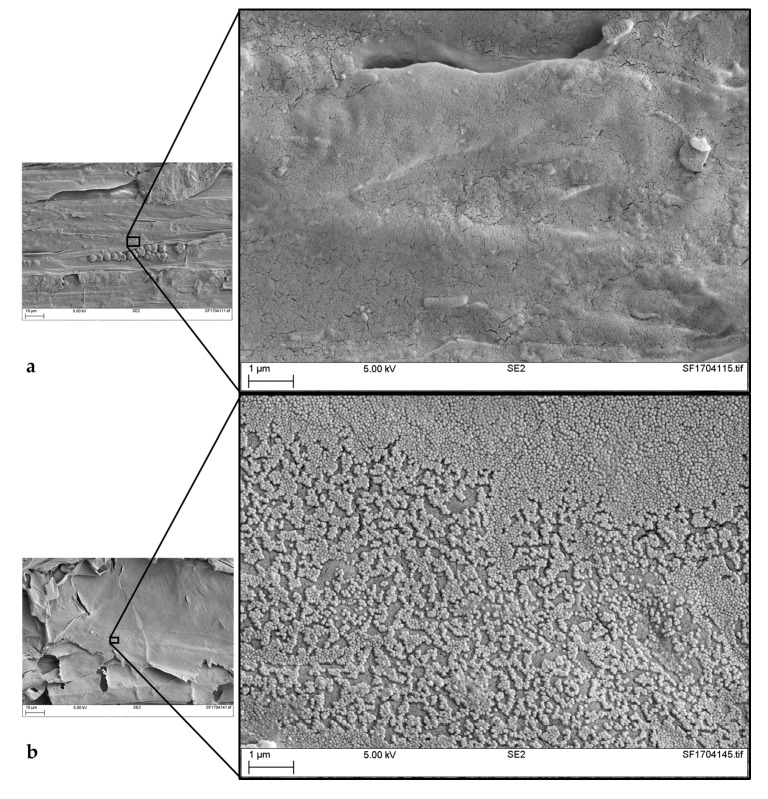

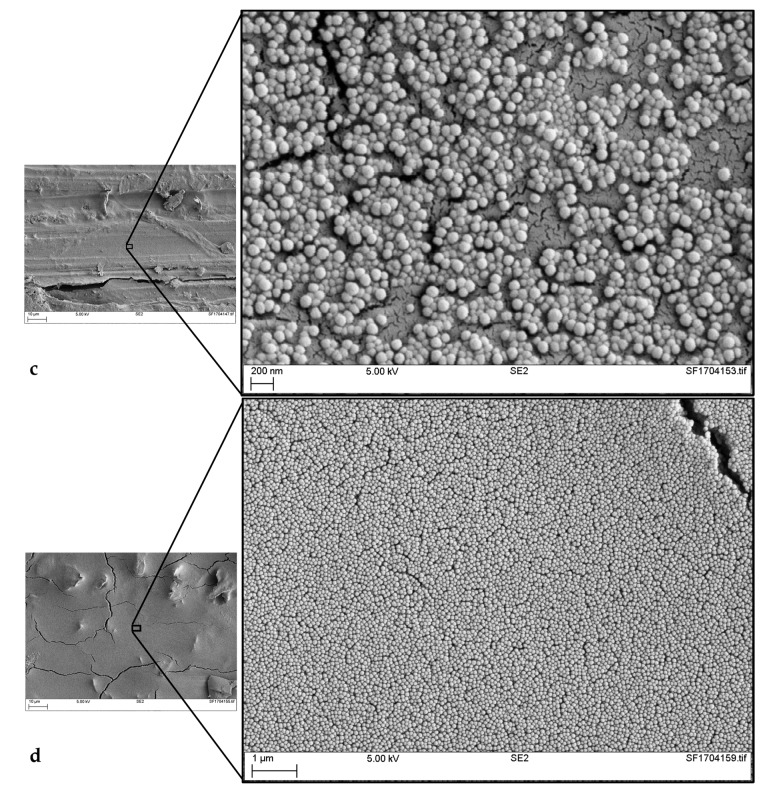
SEM (Scanning Electron Microscopy) images of samples: (**a**) H0-0-0 at ×10,000 magnification; (**b**) H12-1-1 showing a partial coverage at ×10,000 magnification; (**c**) H12-1-2 at ×25,000 magnification; and (**d**) H12-1-3 showing a complete and uniform coverage of the hemp shiv surface at ×10,000 magnification. Courtesy of TWI Ltd.

Only a partial coverage of the surface of the hemp shiv was achieved by single or two layers of silica particles, which corresponds to samples H12-1-1 and H12-1-2 (Figure 3b,c). As further layers were deposited, more complete coverage of the surface of the shiv was achieved, as shown in Figure 3d for the sample H12-1-3. At greater magnification for sample H12-1-2 (Figure 3c), it was possible to verify the size of the particles deposited. Particles with a maximum diameter of 152 nm and a minimum of 90 nm were measured from the image. This fits well with expectations and the assessment of the particles size when in suspension, 120 nm [23].

**Figure 4:**
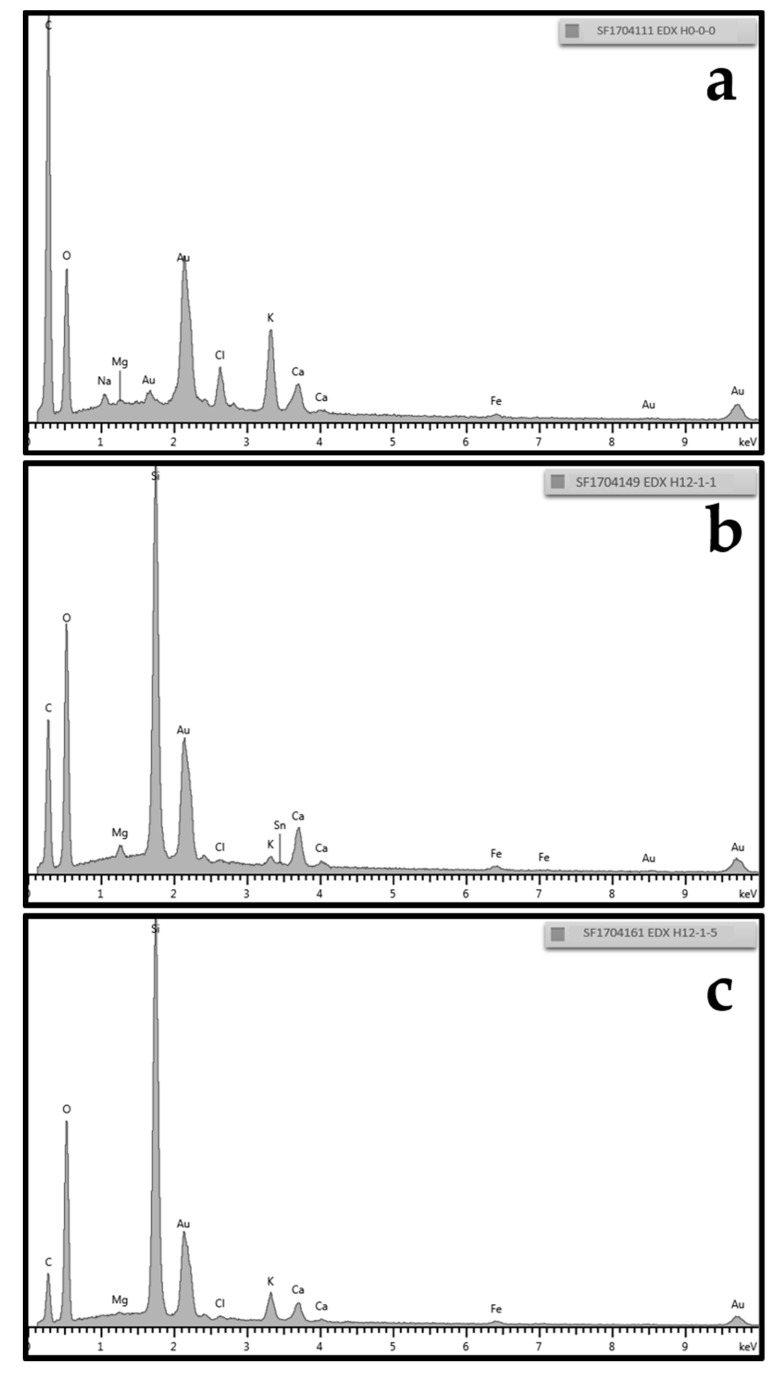
EDX spectra of H0-0-0, H12-1-1, and H12-1-5: (**a**) sample H0-0-0; (**b**) H12-1-1, coated once with 120 nm silica particles; and (**c**) H12-1-5, coated five times. The peaks for Au are due to the gold coating applied for SEM imaging. Courtesy of TWI Ltd.

The EDX spectra of two coated samples and of the untreated hemp shiv are represented in Figure 4. Even though the EDX characterisation method is not quantitative, it is still possible to differentiate which chemical elements are in abundance on the surface of the hemp shiv. The first spectrum shown in Figure 4a belongs to untreated hemp shiv (sample H0-0-0–Figure 3a) that presented a lot of carbon and oxygen, originating from the cellulose of its surface. The same two elements were still present in the second spectrum (Figure 4b), with the addition of silicon from the silica particles. As seen from the SEM image in Figure 3b, the corresponding sample was only coated once with silica particles, and those only partially covered the surface, which is why elements from cellulose could still be seen. Expectedly, the highest peak of the third spectrum (Figure 4c) of sample H12-1-5 shows far greater levels of silicon. In this sample, the signal relating to silica was the most intense compared to those of other elements. This sample was coated five times, and SEM image shows that the coverage by silica particles of the hemp shiv surface was almost complete and uniform for three (H12-1-3–Figure 3d) and more layers of silica.

### 3.3. Water Repellence of Loose Coated Hemp Shiv

**Figure 5:**
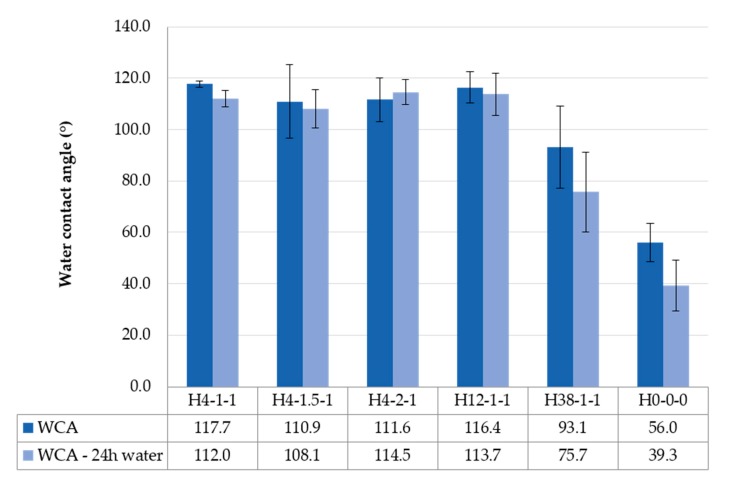
Water contact angle measurements on hemp shiv before (dark shade) and after immersion in a water bath for 24 h (lighter shade). The error bars represent the standard deviation of measurements for each type of sample (several hemp shiv were analysed).

The static WCA was measured for samples H4-1-1, H4-1.5-1, H4-2-1, H12-1-1, H38-1-1, and H0-0-0. The influence of the hydrophobic coating was successfully demonstrated by comparing untreated pieces of hemp shiv (H0-0-0) with different formulations of hydrophobic silica particles applied on hemp shiv. Particles of 45 nm diameter were used in samples H4-x-1, with an increasing amount of alkoxysilane. Doubling the quantity of nPTMS did not have a significant effect on the WCA, as can be seen in Figure 5, which supports the earlier findings on glass slides. Sample H12-1-1 with particles of 120 nm diameter also showed a WCA similar to the other samples, approximately 114° on average across the four treated samples. The WCA for sample H38-1-1 was just over 90°, indicating a less hydrophobic nature than the H4-x-1 and H12-1-1 samples. The particles used for this formulation had a mean diameter of 380 nm, and were applied as a single layer.

The six samples were immersed in a bath of deionised water for 24 h to verify the durability of the coating. Upon drying the samples at 65 °C for 10 min, the WCA was measured again (Figure 5). As expected, the untreated sample showed a significant reduction in the measured WCA, which would suggest that water was still trapped in the structure of the hemp shiv even after drying. Sample H38-1-1 with 380 nm particles also demonstrated a significant reduction in the measured WCA compared to the value prior to immersion. The four samples with particles of 45 and 120 nm diameter maintained their hydrophobic behaviour after immersion in water. The smaller silica particles thus provided an effective barrier to liquid water within and on the hemp shiv.

### 3.4. Increasing the Silica Content and Hydrophobic Period

**Figure 6:**
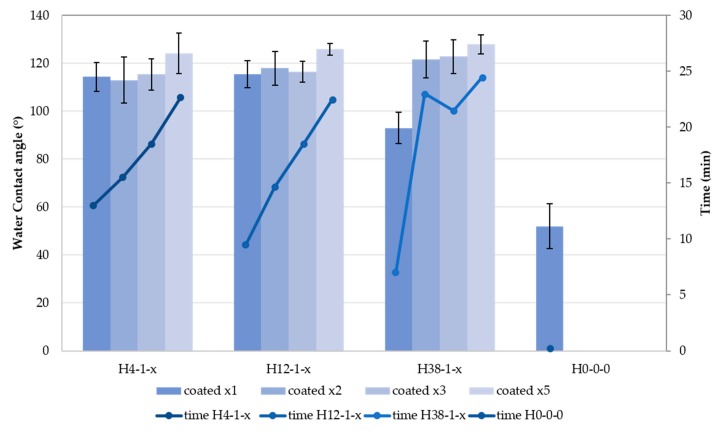
WCA and time measurements for the series of samples H4-1-x, H12-1-x, H38-1-x, and H0-0-0, showing the beneficial effect of the quantity of silica applied onto the hemp shiv on both the WCA, and the period of sustained water repellence.

As was clear from the SEM images (Figure 3), each piece of hemp shiv should be coated more than once to ensure more complete coverage of the surface by silica. It was also proven that increasing the quantity of silica coated increased the WCA of more than 10° (from 115.5° for H12-1-1, coated once, to 125.9° for H12-1-5 coated five times) (Figure 6 and Table 2). Having more silica on the surface provides more barrier effect, thus water is less likely to be absorbed by the hemp shiv.

Cellulose is naturally hydrophilic, which means that, if the hemp shiv are left untreated, liquid water penetrates quickly in the structure of the bio-aggregates. When a droplet of water is deposited on the surface of a piece of hemp shiv, it is absorbed in three to five seconds [29]. However, when analysing coated hemp shiv, hydrophobicity was achieved as the droplet remained on the surface of the shiv with a contact angle greater than 90° for considerably longer. The time for which a single droplet maintained a contact angle greater than 90° with the substrate was monitored for the series of samples H4-1-x, H12-1-x, and H38-1-x. The results are shown in Figure 6. There is a clear correlation between the number of deposited layers on the hemp shiv and the retention of hydrophobic behaviour, which went up to nearly 25 min for sample H38-1-5, and above 20 min for 5 samples. This method has its flaws as some of the water can evaporate during the experiment, but overall it can be a good first assessment of prolonged hydrophobic behaviour of a surface that usually absorbs water. This enhanced resistance to water absorption is due to the barrier effect of the silica. Indeed, the samples coated five times (H4-1-5, H12-1-5, and H38-1-5) were produced so that more inorganic material was deposited between the hemp shiv and the water, therefore preventing the attraction of liquid water by the hydroxyl groups of the cellulose.

### 3.5. Humidity Tests

**Figure 7:**
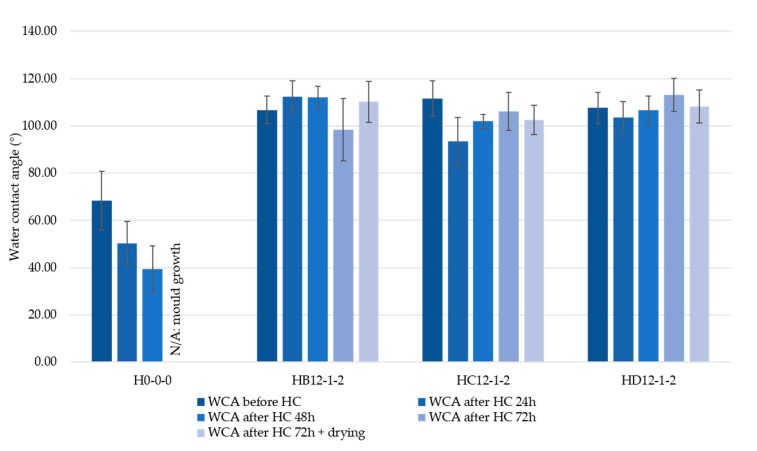
Humidity chamber test (35 °C and 90% RH). The measurements were executed every 24 h until signs of mould growth were visible on at least one sample, and after drying at room temperature for five days. Only very little variation of WCA during the experiment for the coated samples was shown, and mould was prevented from developing.

The bio-materials analysed in this study are aimed to be used as insulation as the core of panels in walls. The reason for this is the ability of the hemp shiv to buffer moisture thanks to their microporosity [3]. This breathable material then allows water vapour to permeate, and, when coated with silica particles, prevents absorption of liquid water. When vapour condensates within the panel, it is important that the bio-materials continue to repel liquid water. Moreover, bio-materials are subject to natural biological decay, which is usually triggered by a humid and warm environment. To assess the effect of humidity on the treated bio-aggregates, samples H0-0-0, HB12-1-2, HC12-1-2, and HD12-1-2 were placed in a humidity chamber at 35 °C and 90% of relative humidity. The three treated samples were coated with the same formulation of hydrophobic silica particles, but were deposited on three different grades of hemp shiv. As the performance of each type of coating has been assessed in the previous sections, verifying the water repellence on bigger pieces of hemp shiv can give insightful information on the influence (or lack thereof) of the substrate. The WCA of the loose materials was measured at regular intervals. The change in repellence of the samples throughout this experiment is shown in Figure 7. Untreated hemp shiv (sample H0-0-0) became more hydrophilic the longer they were exposed to humidity, with the WCA decreasing from 68° to 39° in 48 h of exposure. The absence of data for 72 h of exposure is due to the development of fungal growths which led to the sample being discarded. This period of exposure marked the termination of the test. On the other hand, coated hemp shiv consistently maintained a WCA of 115° on average across the different samples, all throughout the three days of humidity exposure. Upon termination of the experiment and after drying the samples, the final WCA remained consistent with the values obtained during the humidity exposure, with no sign of microbial growth. The presence of silica particles on hemp shiv again proved to act as a barrier to liquid water, but also to prevent mould from developing.

## 4. Discussion

Due to their microporosity and cellulosic content, hemp shiv are hydrophilic and have a very high potential of water uptake, as they can absorb up to three times their own dry weight [7]. Coating the hemp shiv with hydrophobic silica particles is potentially beneficial in two ways. The coating may act as a barrier to liquid water, so droplets would just sit on the surface of the hemp shiv; however, if the silica particles are small enough, they would not block the primary pores of the hemp shiv and water vapour could can still permeate allowing the hemp shiv to retain its buffer properties.

**Figure 8:**
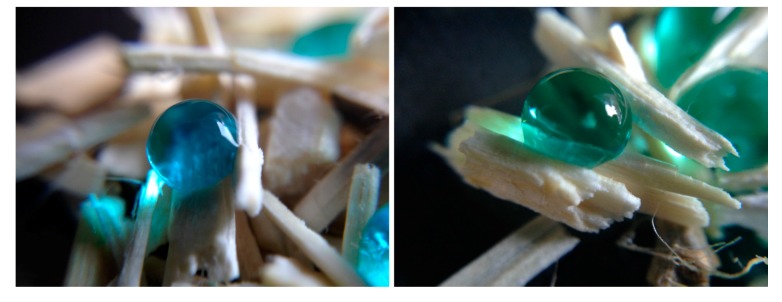
Coloured water droplets onto hemp shiv H12-1-5, with a WCA > 125°. Courtesy of TWI Ltd.

We demonstrated in this study that the more functionalised silica was present on the surface, the greater the water contact angle was. Imaging with SEM techniques demonstrated that several layers of coating led to a more complete and uniform coverage of the hemp shiv. The additional layers also increased the time it took for a droplet of water to be completely absorbed, from five seconds to beyond 25 min. This demonstrated that functionalised silica particles could be used as a hydrophobic treatment for bio-materials, as they provided an effective barrier to water (as illustrated in Figure 8).

The presence of inorganic material between the shiv and the environment protected it from the effects biodegradation, as well as reduced the potential attraction of water by the hydroxyl groups of the cellulose. The bond between cellulose and silica was strong enough to withstand exposure to humid conditions and immersion in water, when small particles (45 or 120 nm diameter) were applied on the hemp shiv.

The silica particles also brought microroughness to the surface of the shiv. This characteristic was demonstrated by other researchers [20] by the means of a 3D optical profilometer. Using such characterisation technique could be valuable for quantifying how much silica was effectively deposited onto the bio-aggregates after being coated multiple times.

## 5. Conclusions

In this study, a hydrophobic treatment was applied onto hemp shiv that are starting to be used in insulation for buildings. When functionalised silica particles were coated several times onto the hemp shiv, a uniform and complete coverage of the surface was achieved. We successfully demonstrated that such treatment provided hemp shiv with extensive water repellence: the hydrophilic character of the untreated hemp shiv was modified to durably hydrophobic once the bio-materials were coated with functionalised silica particles. Mould growth was delayed when exposed to humidity, whilst the liquid water repellent property of the treated hemp shiv was maintained despite the humid conditions. The treatment developed in this study could be a viable solution for use on bio-materials that need to repel liquid water, whilst preserving the integrity of the insulation panel in common environmental conditions. There are of course still further tests to be carried out before adopting this treatment in the construction sector, such as additional moisture tests, mechanical tests, biodegradation tests, etc. However, the promising results described here give a good first assessment of feasibility. Before being fully adopted in the construction sector, such treatment and materials will have to be fully tested according to building codes. Considering the only recent use of bio-materials, new standards and test methods should be developed for those specific materials.

## Figures and Tables

**Table 1:** Description of the samples with the characteristics of each treatment, where sample nomenclature refers to firstly the substrate, then silica particle size, then functionalisation level, and finally the number of coating layers.

Sample	Substrate	Treatment	
		Functionalisation Level	Coated × Times
H0-0-0	hemp shiv G7	none	
H4-1-1	hemp shiv G7	1.00	1
H4-1-2	hemp shiv G7	1.00	2
H4-1-3	hemp shiv G7	1.00	3
H4-1-5	hemp shiv G7	1.00	5
H12-1-1	hemp shiv G7	1.00	1
H12-1-2	hemp shiv G7	1.00	2
H12-1-3	hemp shiv G7	1.00	3
H12-1-5	hemp shiv G7	1.00	5
H38-1-1	hemp shiv G7	1.00	1
H38-1-2	hemp shiv G7	1.00	2
H38-1-3	hemp shiv G7	1.00	3
H38-1-5	hemp shiv G7	1.00	5
H4-1.5-1	hemp shiv G7	1.50	1
H4-2-1	hemp shiv G7	2.00	1
HB12-1-2	hemp shiv G8	1.00	2
HC12-1-2	hemp shiv Biofibat	1.00	2
HD12-1-2	hemp shiv Isofin	1.00	2

**Table 2:** Summary of results, after Figure 6.

		H4-1-x	H12-1-x	H38-1-x	H0-0-0
coated × 1	WCA (°)	114.4	115.5	93.1	52.0
	time to 90° (min)	13.0	9.5	7.0	0.1
coated × 2	WCA (°)	113.0	117.9	121.6	
	time to 90° (min)	15.5	14.7	23.0	
coated × 3	WCA (°)	115.4	116.5	122.8	
	time to 90° (min)	18.5	18.5	21.5	
coated × 5	WCA (°)	124.2	125.9	128.0	
	time to 90° (min)	22.7	22.5	24.5	
